# Meta-Analysis of Peripheral Blood Apolipoprotein E Levels in Alzheimer’s Disease

**DOI:** 10.1371/journal.pone.0089041

**Published:** 2014-02-18

**Authors:** Chong Wang, Jin-Tai Yu, Hui-Fu Wang, Teng Jiang, Chen-Chen Tan, Xiang-Fei Meng, Holly D. Soares, Lan Tan

**Affiliations:** 1 Department of Neurology, Qingdao Municipal Hospital, School of Medicine, Qingdao University, Qingdao, Shandong, China; 2 Department of Neurology, Qingdao Municipal Hospital, Nanjing Medical University, Nanjing, Jiangsu, China; 3 College of Medicine and Pharmaceutics, Ocean University of China, Qingdao, Shandong, China; 4 Bristol-Myers Squibb, Wallingford Center, Connecticut, United States of America; University of Florida, United States of America

## Abstract

**Background:**

Peripheral blood Apolipoprotein E (ApoE) levels have been proposed as biomarkers of Alzheimer’s disease (AD), but previous studies on levels of ApoE in blood remain inconsistent. This meta-analysis was designed to re-examine the potential role of peripheral ApoE in AD diagnosis and its potential value as a candidate biomarker.

**Methods:**

We conducted a systematic literature search of MEDLINE, EMBASE, the Cochrane library, and BIOSIS previews for case-control studies measuring ApoE levels in serum or plasma from AD subjects and healthy controls. The pooled weighted mean difference (WMD) and 95% confidence interval (CI) were used to estimate the association between ApoE levels and AD risk.

**Results:**

Eight studies with a total of 2250 controls and 1498 AD cases were identified and analyzed. The pooled WMD from a random-effect model of AD participants compared with the healthy controls was −5.59 mg/l (95% CI: [−8.12, −3.06]). The overall pattern in WMD was not varied by characteristics of study, including age**,** country, assay method, publication year, and sample type.

**Conclusions:**

Our meta-analysis supports a lowered level of blood ApoE in AD patients, and indicates its potential value as an important risk factor for AD. Further investigation employing standardized assay for ApoE measurement are still warranted to uncover the precise role of ApoE in the pathophysiology of AD.

## Introduction

Alzheimer’s disease (AD) is the most common type of dementia characterized by a profound loss of memory, progressive decline in cognition, and changes in personality [1]. It is estimated that more than 5.2 million people were living with AD in USA by 2013 [2]. However, the pathogenesis of AD remains elusive thus far. Generally, AD is diagnosed on the basis of clinical criteria such as loss of cognitive function. However, by the time the patient is diagnosed with AD, the disease has been progressing for many years [3]. There is currently no known cure in reversing cognitive decline, with treatments generally aimed at slowing disease progression [4]. Thus it is of great importance to diagnose AD at an early stage and perform effective interventions before cognitive symptoms emerge. Although some cerebrospinal fluid (CSF) biomarkers and amyloid imaging have been established to detect AD pre-clinically [5], [6], these are not suitable for large scale screening programs in consideration of the invasiveness and a host of comorbidities. Taking into account these facts, finding proteins from peripheral blood samples that could identify AD may be useful for pre-symptomatic. To date, sufficient evidence has demonstrated that the Apolipoprotein E (*APOE*) ε4 allele is a well-established risk factor for sporadic AD [7], [8]. Besides the genetic polymorphism, ApoE levels in both plasma and serum may reflect disease status as well. ApoE is a protein involved in transport of lipids and it is also an important antioxidant [9]. As understanding of ApoE has evolved, existing evidence in neurobiology suggests that ApoE may play a central, if not direct, role in the pathogenesis of AD [10]. Therefore, peripheral blood ApoE levels may represent a significant clinical diagnostic biomarker for the future development of an early AD blood diagnostic.

Given the range of methods used and variable sample sizes of the studies for assessing blood ApoE levels, the changes of peripheral ApoE levels in AD patients vs. controls have been a subject of contention primarily because of inconsistent findings from previous studies. While the majority of studies reported ApoE levels in AD patients were lower than healthy controls, some studies showed no significant change or even a slight increase of ApoE levels. To our knowledge, no meta-analysis of such studies has been conducted on the association between peripheral ApoE level and AD. Given these reasons, a meta-analysis could be helpful in resolving the controversial question of whether peripheral ApoE level is of value in the diagnosis of AD.

## Methods

### Data sources

We conducted a systematic literature search of MEDLINE, EMBASE, the Cochrane library, and BIOSIS previews for studies published in the period from January 1995 to July 2013 using terms including “serum”, “plasma”, “Apolipoprotein E”, “Alzheimer”, and “dementia”, combined with Boolean operators as appropriate. There were no language restrictions, and translation was obtained as necessary. Additional studies were obtained from the reference lists of identified studies.

### Study selection

We assessed studies appropriateness and included studies if they meet the following criteria: (1) Case-control studies design; (2) Diagnosis of AD was performed according to the National Institute of Neurological Disorders and Stroke-Alzheimer Diseases and Related Disorders Association Working Group criteria (NINCDS–ADRDA); (3) Data of the plasma or serum ApoE levels are available in the report or can be obtained from the corresponding author. We excluded reviews, letters without original data, editorials, and papers focused on familial AD. Moreover, when there was more than one publication from the same population, only data from the most comprehensive report were included in the meta-analysis and the remaining were excluded.

### Data extraction and quality assessment

Two reviewers independently performed the search, reviewing all articles and collecting data. The mean, standard deviation (SD) or Standard Error (SE) on plasma or serum ApoE levels were extracted. When both crude and adjusted ApoE levels were provided, we used the most fully adjusted ApoE levels for all the included studies. Because the underlying units of measurement varied from study to study, all units were converted to mg/l. From each included study, we also recorded the following data when available: publication year, country of study origin, criteria for AD diagnosis, sample size, mean age, percentage of females in groups, and statistical adjustments for confounding factors.

We assessed how ApoE levels were measured with the following data: type of sample; assay method; laboratory or kit used; collection, process, and storage of sample; blinding of laboratory personnel; and the use of quality control sample. The study quality was assessed independently by two reviewers with consideration of the following aspects followed the Newcastle-Ottawa quality assessment scale case control studies [11]: 1) the Selection; 2) the Comparability; 3) the Exposure. We identify ‘high’ quality choices with a ‘star’. A study can be awarded a maximum of one star for each numbered item within the Selection and Exposure categories. A maximum of two stars can be given for Comparability. Studies with a score equal to or higher than seven were considered to be high quality.

### Data synthesis

All statistical analyses were performed using weighted mean difference (WMD) methodology in Review Manager (version 5.2.3 for Windows Copenhagen: The Nordic Cochrane Centre, The Cochrane Collaboration, 2012). In our studies, WMD is the pooled difference between AD groups and control groups on mean values across a group of studies using the same scale of measurement for the outcome. We used the 95% confidence interval (CI) to gauge the precision of the summary estimates. A random-effects model or fixed-effects model was used to calculate pooled WMD in the presence or absence of heterogeneity, respectively.

We performed subgroup analysis to explore the association between characteristic of studies and their results. The following study characteristics were examined with cut points for binary variable specifications selected to achieve as close as possible balanced distributions, average age of study participants (≥ 75 or <75) country (Europe or others), assay method (ELISA or immunoturbidimetry), publication year (≤2001 or >2001) and sample type (plasma or serum). We also performed sensitivity analyses to assess the influence of individual studies on the pooled WMD.

Publication bias was investigated using funnel plots, with a roughly symmetrical distributed on either side of the summary estimate suggesting a lack of bias. Overall heterogeneity was assessed using the Cochran Q (P value was greater than 0.10 on the Q test, which reflects a lack of heterogeneity among studies) and I^2^ (values of more than 50% as “considerable heterogeneity”) [12], [13].

## Results

### Literature search and characteristics of included studies

**Figure 1:**
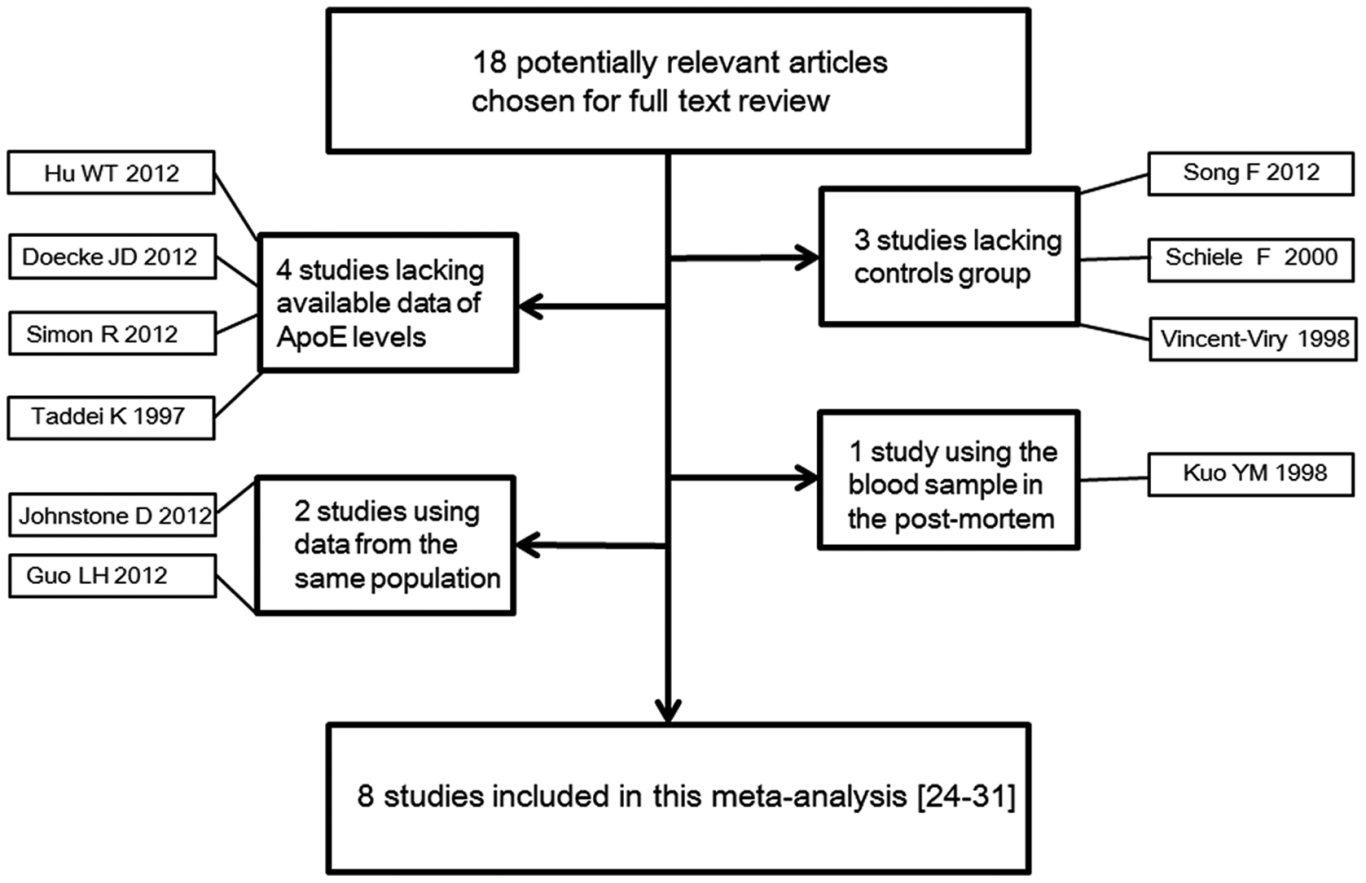
Flow diagram of the study selection process. ApoE, apolipoprotein E.

After the application of search strategy, we found that a total of 18 relevant articles appeared to fulfill the inclusion criteria [14]–[31]. After reviewing the full texts, 10 additional studies were excluded, leaving 8 trials [24]–[31], published between 1998 and 2012, that met our selection criteria and had accessible plasma or serum ApoE concentration information to study the association between ApoE levels and AD (Figure 1). The pooled subjects included a total of 2250 controls and 1498 AD cases in Europe (5 studies), Asia (1 studies) and North American (2 studies). The age range of all the subjects ranged from 65 to 86 y. Most studies included both males and females in AD cases and controls. The adjustment for confounding factors was described in 5 studies. Five studies provided ApoE levels in AD and healthy controls, stratified according to the *APOE* genotypes. Data details of the included studies are presented in Tables 1 and Table 2.

**Table 1:** Characteristics of the included studies for the meta-analysis.

Study (country)	Diagnosis(n)		Diagnostic criteria	Mean age (y)	Female %	ApoE levels		P value	Adjustment for confounding	NOS
						original ApoE levels	converted ApoE levels			
**Holly D. Soares (2012)**	control	58	NINCDS–ADRDA	75±6	48.30%	75.89±26.94 µg/ml^a^	75.89±26.94 mg/l	NR	NR	7
**(** ***North American*** **)**	AD	112		75±8	42.00%	56.77±26.83 µg/ml^a^	56.77±26.83 mg/l			
**V.B. Gupta (2011)**	control	365	NINCDS-ADRDA	70±7	NR	15.43±2.66 mg/dl	154.3± 26.6 mg/l	P = 0.044	age and genotypes	8
**(** ***North American*** **)**	AD	199		78±8	NR	14.23 ±2.63 mg/dl	142.3± 26.3 mg/l			
**Francesco Panza (2003)**	control	45	NINCDS–ADRDA	65.8±11.6	71.10%	44.0±7.0 mg/l	44.0±7.0 mg/l	P = 0.23	NR	9
**(** ***Europe*** **)**	AD	49		71.6±9.3	69.40%	39.0±10.0 mg/l	39.0±10.0 mg/l			
**G. Roks (2002)**	control	247	NINCDS–ADRDA	74.7±3.6	85%	2.7±1.6 mg/dl	27±16 mg/l	P<0.01	age gender locus	8
**(** ***Europe*** **)**	AD	360		82.4±7.1	76%	2.3±1.6 mg/dl	23±16 mg/l			
**Fan Ping (2001)**	control	60	NINCDS–ADRDA	73.2±3.4	56.67%	49±18 mg/l	49±18 mg/l	P<0.05	NR	9
**(** ***Asia*** **)**	AD	75		78.9±7.9	62.67%	40±19 mg/l	40±19 mg/l			
**Gérard Siest (2000)**	control	429	NINCDS–ADRDA	71.0±9.1	59.70%	47.4±13.2 mg/l	47.4±13.2 mg/l	P<0.001	age, sex and genotypes	7
**(** ***Europe*** **)**	AD	489	DMS-IV	74.5±8.6	64.10%	43.7±13.0 mg/l	43.7±13.0 mg/l			
**R.Scacchi (1999)**	control	156	NINCDS-ADRDA	83.8±3.2	58.30%	4.43±1.43 mg/dl	44.3±14.3 mg/l	NR	age and genotypes	7
**(** ***Europe*** **)**	AD	85		86.1±3.8	76.50%	4.45±1.51 mg/dl	44.5±15.1 mg/l			
**Arijen J.C. (1998)**	control	890	NINCDS–ADRDA	68.2±7.2	59%	0.83±0.40 µmol/l	28.3± 13.7 mg/l^b^	P<0.05	age, sex, BMI, total protein	8
**(** ***Europe*** **)**	AD	129		84.1±6.5	73%	0.75±0.35 µmol/l	25.6± 12.0 mg/l^b^		and albumin level	

Abbreviations: AD, Alzheimer’s disease; ApoE, Apolipoprotein E; NR, not reported; NINCDS–ADRDA, the National Institute of Neurological Disorders and Stroke–Alzheimer Diseases and Related Disorders Association Working Group criteria; DMS-IV, Diagnostic and statistical manual of mental disorders 4th Edition; BMI: body mass index; NOS, Newcastle-Ottawa Scale.

Data are presented as Mean ± SD;

a: Mean ± SD values were obtained from corresponding authors.

b: µmol/L values were converted using molecular weight values of 34145 g/mol for ApoE.

**Table 2:** ApoE levels in AD and healthy controls, stratified according to the *APOE* genotypes.

Study		ApoE levels in AD and controls (n), mg/l					
		ε2/ε2	ε3/ε2	ε4/ε2	ε3/ε3	ε4/ε3	ε4/ε4
**Arijen J.C.^b^ (1998)**	control	60.1± 22.9(7)	39.9±16.4(130)	37.9±15.0(18)	27.7±11.6(498)	21.9±8.9(221)	14.7±4.4(16)
	AD	77.5(1)	36.9±14.0(16)	33.5±10.6(3)	24.6±9.9(69)	20.5±6.1(33)	13.3±7.9(7)
**R.Scacchi (1999)**	control	-	50.1±19.2 (12)	31.7 (1)	44.0±13.5 (123)	42.9±12.1 (16)	-
	AD	-	70.7±27.3 (7)	57.3 (1)	43.3±10.1 (51)	39.2±7.8 (22)	24.3±7.5 (2)
**Francesco Panza (2003)**	control	NR	NR	NR	43.2±2.1(17)	NR	NR
	AD				40.5±1.5(32)		
**V.B.Gupta (2011)**	control	NR	NR	NR	161.31±25.3(207)	142.93±23.2(82)	NR
	AD				146.21±28.4(70)	142.02±23.1(93)	
**Holly D. Soares^a^ (2012)**	control	-	90.00±24.72 (17)	144 (1)	68.94±23.64 (36)	61.50±22.04 (4)	-
	AD	-	49.00(1)	61.5±17.68 (2)	74.71±33.55 (35)	51.31±16.61 (51)	41.52±20.53 (23)

Abbreviations: AD, Alzheimer’s disease; ApoE, Apolipoprotein E; NR, not reported.

Data are presented as Mean ± SD; all ApoE values were expressed in mg/l;

a:.Mean ± SD values were obtained from corresponding authors.

b:µmol/L values were converted using molecular weight values of 34145 g/mol for ApoE.

### Quality of reporting on ApoE assay and quality assessment of included studies

Of all included studies, 4 measured the ApoE levels in plasma-based sample [27−29], , and the others in serum-based sample [24−26], (Table 3). The collection, process and storage of sample were described in sufficient details in 5 studies. Five studies collected blood samples from fasting participants [24−26], [28], . Only three studies reported the blinding of laboratory personnel in the ApoE assay. It is important to note that the use of quality control sample was rarely reported in all studies. Quality assessment showed that the NOS score of each study was not less than 7, indicating that the methodological quality was generally good.

**Table 3:** Quality of reporting on ApoE assay in 8 included studies.

Study (publication year)	Sample	Assay method	Laboratory or Kit Used	Collection process and storage of sample	Blinding of laboratory personnel	Use of quality control sample
**Arijen J.C.(1998)**	serum	sandwich ELISA	NR	venipuncture; non-fasting	yes	NR
**R.Scacchi (1999)**	plasma	immunoturbidimetry	Eichen Chemical, Tokyo, Japan	NR	NR	NR
**Gérard Siest (2000)**	serum	immunoturbidimetry	Daiichi Pure Chemical, Tokyo, Japan	venipuncture; EDTA vacutube; overnight fasting	NR	NR
**Fan Ping (2001)**	serum	immunoturbidimetry	NR	venipuncture; fasting; −30°C	NR	NR
**G. Roks (2002)**	plasma	sandwich ELISA	NR	non-fasting	NR	NR
**Francesco Panza (2003)**	serum	nephelometry	Nephelometer 100 Analyzer, Behring, Germany	fasting	NR	NR
**V.B.Gupta (2011)**	plasma	sandwich ELISA	MBL Co., Ltd	fasting; EDTA	yes	NR
**Holly D. Soares (2012)**	plasma	ELISA	RBM Inc. Austin, TX, USA	overnight-fasting; EDTA	yes	NR

Abbreviations: ELISA, enzyme-linked immunosorbent assay; EDTA, ethylenediaminetetraacetic acid; NR, not reported.

### ApoE levels between AD cases and controls

**Figure 2:**
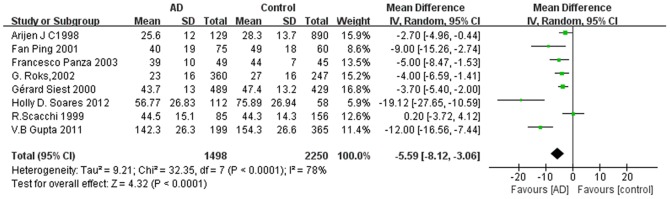
Forest plots for ApoE levels in AD and healthy controls in included studies. AD, Alzheimer’s disease; SD, standard deviation; CI, confidence interval.

**Figure 3:**
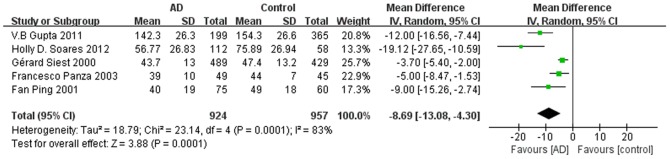
Forest plots for ApoE levels in AD and healthy controls controlling for fasting. AD, Alzheimer’s disease; SD, standard deviation; CI, confidence interval.

Of eight included studies, six studies reported that ApoE levels were significantly lower in AD patients than controls, whereas two studies found no significant difference. Combined analysis of the relationship between the peripheral blood ApoE level and AD was shown in forest plots (Figure 2). Compared to healthy controls, AD subjects had a lower ApoE level. The pooled WMD from a random-effects model was −5.59 mg/l (95% CI: [−8.12, −3.06]; I^2^ = 78%; Cochran’s Q = 32.35; P<0.0001); the test for overall effect: Z = 4.32; p<0.0001. To eliminate the influence of non-fasting on blood ApoE levels, we also performed an additional analysis of the 5 studies that collected blood sample from fasting participants. The pooled WMD was −8.69 mg/l (95% CI: [−13.08, −4.3]; I^2^ = 83%; Cochran’s Q  = 23.14; P = 0.0001) (Figure 3).

Within all subgroups, the pooled WMD was consistently negative, indicating that ApoE level was lower in patients with AD than healthy controls (Table 4). The pooled WMD was more negative in studies conducted among non-Europeans (WMD: −12.59 [−17.37, −7.82]). On the contrary, studies published in 2001 or earlier had a less negative pooled WMD (−3.15 [−5.36, −0.94]) compared to other subgroups. The overall pattern in WMD did not vary by age**,** country, assay method, publication year and sample type. Moreover, no heterogeneity was found in European group (I^2^ = 16%, P = 0.31), non-European group (I^2^ = 43%, P = 0.17), and serum-based sample group (I^2^ = 26%, P = 0.26). Of course, there was significant unexplained between-study heterogeneity within the subgroups in spite of stratified analyses.

**Table 4:** Stratified analysis of ApoE levels by study characteristics.

Characteristics	Number of studies	Pooled WMD^a^	Within-stratum heterogeneity
**Age, y**			
≥ 75	4	−6.87 [−12.75, −1.00]	I^2^ = 84%, P = 0.0003
< 75	4	−5.25 [−8.14, −2.35]	I^2^ = 78%, P = 0.004
**Country**			
Europe	5	−3.31 (−4.54, −2.08)	I^2^ = 16%, P = 0.31
others	3	−12.59 [−17.37, −7.82]	I^2^ = 43%, P = 0.17
Assay method			
ELISA	4	−8.14 [−13.26, −3.03]	I^2^ = 88%, P<0.0001
immunoturbidimetry	4	−3.82 [−6.47, −1.17]	I^2^ = 58%, P = 0.07
**Publication year**			
≤2001	4	−3.15 [−5.36, −0.94]	I^2^ = 55%, P = 0.08
>2001	4	−8.88−14.00, −3.77]	I^2^ = 84%, P = 0.0004
**Sample**			
plasma	4	−7.88 [−14.30, −1.45]	I^2^ = 89%, P<0.00001
serum	4	−3.91 [−5.48, −2.34]	I^2^ = 26%, P = 0.26

WMD: weight mean difference; CI: confidence interval; ELISA: enzyme linked immunosorbent assay.

**Figure 4:**
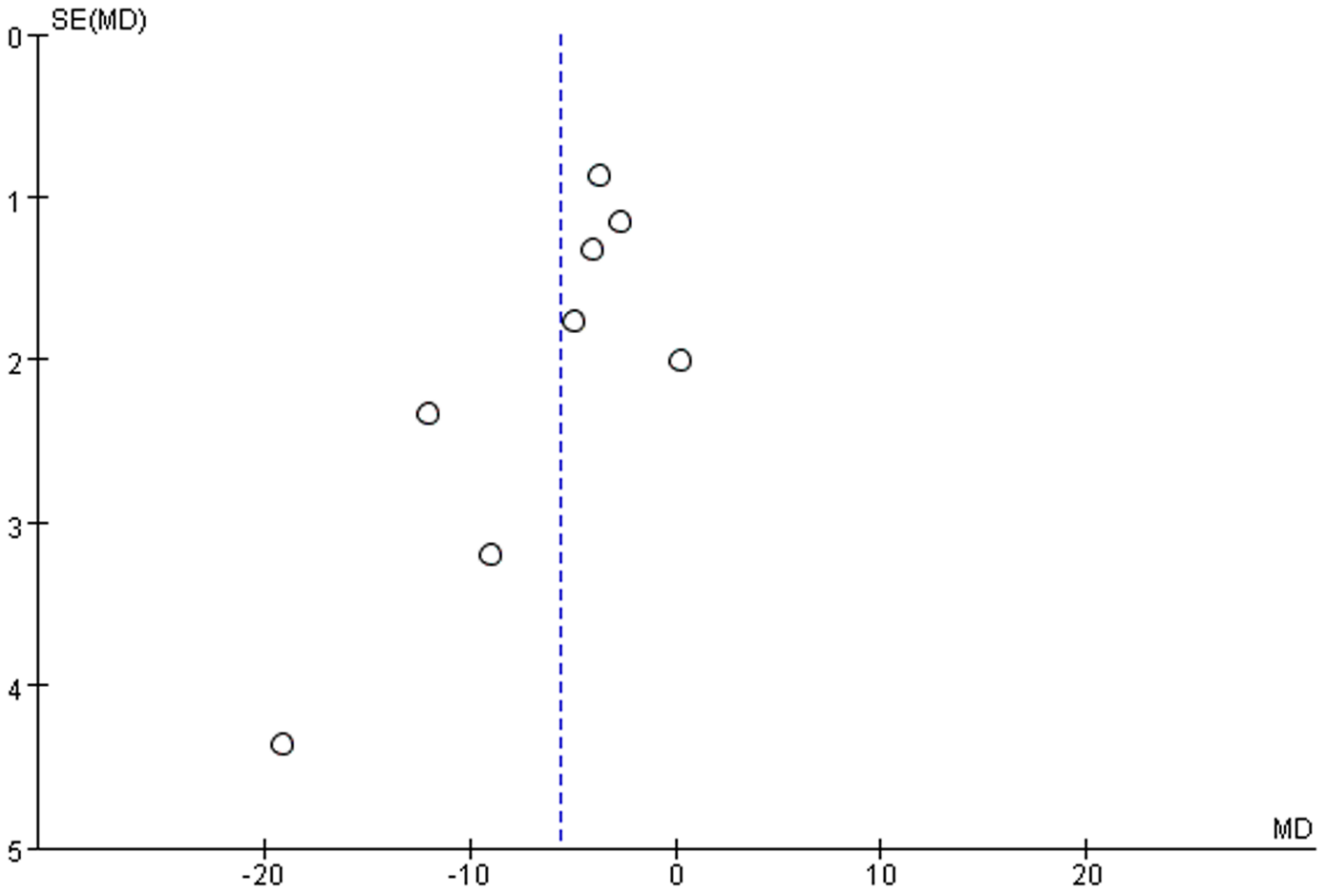
Funnel plots for ApoE levels in AD and healthy controls in included studies. Vertical dashed lines represent the summary weighted mean difference (WMD).

As sensitivity analyses, we excluded 1 study at a time to assess the stability of the results. There was no significant change in the pooled WMD or 95%CI on excluding any of the studies (WMD lied between −4.80 and −6.38). The study of V.B.Gupta 2011 [28] and Holly D. Soares 2012 [31] which were the only two published after 2003 seemed to have a larger influence, but the result did not change materially after exclusion of these two studies (WMD: −3.54; 95%CI: [−5.00, −2.08]), which suggested that the overall results of this meta-analysis were statistically robust. In addition, the heterogeneity was effectively removed after excluding these two studies (Cochran Q = 7.79; P = 0.17; I^2^ = 36%). Visual inspection of the funnel plot (Figure 4) indicated asymmetrical distribution of WMD, suggesting publication bias.

**Figure 5:**
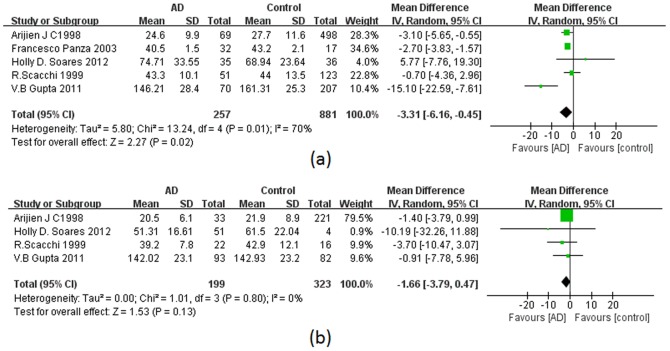
Forest plots for ApoE levels in ε3/ε3 and ε3/ε4 carriers. (a) Forest plots for ApoE levels in ε3/ε3 carriers. (b) Forest plots for ApoE levels in ε3/ε4 carriers. AD, Alzheimer’s disease; SD, standard deviation; CI, confidence interval.

Five studies reported the ApoE concentration for AD patients and normal controls stratified according to the *APOE* genotypes. Because of lacking sufficient numbers of subjects from included studies, we just performed the analysis in ε3/ε3 and ε3/ε4 separately. In ε3/ε3 carriers, the WMD was negative as well (−3.31 [−6.16, −0.45]; P = 0.01). However, we found no significant association between ApoE and AD among ε3/ε4 carriers (−1.66 [−3.79, 0.47]; P = 0.80). (Figure 5)

## Discussion

Compared with CSF, blood analysis can be accessed with minimal discomfort to the subjects, which is more feasible for large populations screening. Therefore, a series of studies have reported blood-biomarkers of AD [32]–[34]. Given the inconsistent reports of previous studies, we examined the potential role of peripheral ApoE in AD pathology and as a strong candidate risk factor. The result of our meta-analysis was unequivocal, with AD patients showing a lower ApoE level than healthy controls.

Our meta-analysis adds to the existing evidence that lower peripheral blood ApoE level is significantly associated with AD and may be an important risk factor for AD. To clarify the sources of heterogeneity and make a more comprehensive analysis, we performed the subgroup analyses. The corresponding pooled WMDs were not materially altered in all subgroups, lending support to a pathogenetic role of ApoE in AD. In the subgroup based on country, the heterogeneity was effectively removed, with more negative WMD found among the non-European group. There was also a huge gap of corresponding WMDs (−3.31 [−4.54, −2.08] vs. −12.59 [−17.37, −7.82]) between European and non-European, suggesting that the variances in the population characteristics and geographic locale seem to play important roles in ApoE levels. This difference may be also induced by various environmental factors, genetic factors, lifestyle and economic status. It is worth to note that sample type is another important confounding factor. Different sample types selected by different laboratory will inevitably lead to different results. In our meta-analysis, plasma-based samples had a more negative WMD than serum-based samples (−7.88 [−14.30, −1.45] vs. −3.91 [−5.48, −2.34]). The difference in results between a plasma-based study and a serum-based study may be due to protein-protein interactions between analytes and clotting factors, or interaction between analytes and additives such as EDTA and potentially different plastic used in the selection, storage of plasma and serum tubes. Furthermore, due to the strict inclusion criteria, we included just two studies published after 2003. These two studies were exactly identified as the main contributors to heterogeneity through the sensitivity analysis.

Taking account of *APOE* genotypes, several previous studies have shown significant associations between peripheral ApoE levels and *APOE* genotypes with a gradient of ApoE levels (ε2>ε3>ε4) [20], [35]. In our included studies, V.B.Gupta 2011 and Holly D. Soares 2012 reported the data of plasma ApoE levels based on *APOE* allelic status in all participants irrespective of disease stage [28], [31]. Both of them also observed a significant trend in decrease of plasma ApoE levels from ε2 to ε4 carriers, with ε4/ε4 carriers expressing the lowest ApoE levels in the peripheral. In both AD patients and controls, ε2 carriers were found to be associated with higher ApoE levels whereas ε4 carriers with lower levels. The different ApoE levels between controls and AD patients may be induced by the different distribution of the genotypes. The association of ε4 genotype with lower ApoE levels may explain in part why ε4 carriers are at higher risk of developing AD. However, in Arijen J.C 1998 study, serum ApoE level was still lower in AD patients than in the controls for each *APOE* genotype, while it was not statistically significant [30]. The exception was the ε2/ε2 carriers with only one AD case. To find out the influence of *APOE* genotypes on peripheral ApoE level, we performed a meta-analysis stratified according to the *APOE* genotypes. From our analysis of ε3/ε3 carriers, the lower ApoE levels in AD patients remained significant in the absence of *APOE* ε4, suggesting that lower ApoE levels in AD is irrespective of *APOE* genotypes to some extent. Lower ApoE levels appear to be an additional risk factor in AD independently of ε4. In line with this notion, Fumihiko Yasuno et al. found that lower plasma ApoE concentration had lower cognitive scores in both ε4- and ε4+ groups in elderly individuals of 3-year follow-up [36]. Of course, we found no significant association between lower ApoE levels and AD in ε3/ε4 carriers. We are not certain about the importance of considering a subject’s *APOE* genotype if lower ApoE level is to be used as a diagnostic tool. Unfortunately, we could not obtain more information from all studies on the frequency of *APOE* genotype in our meta-analysis. Therefore, further evaluation of potential interactions was limited by the lack of original data from included studies, and more investigations are still warranted to find various ApoE levels associated to different genotypes in AD patients.

Because of considerable between-study heterogeneity and variable quality of ApoE assay, our meta-analysis should be interpreted with caution. In fact, studies included in our meta-analysis varied widely with respect to ApoE assay, which may affect the consistency of outcomes between laboratories. However, there was no standardization of sample handing protocols in ApoE measurement. The differences in the assay procedure may cause variation of ApoE levels measured in different studies. The V.B.Gupta 2011 study [28] reported highest mean values of ApoE levels, which is almost 5 fold higher than the Arijen J.C. 1998 study [30]. Therefore, all studies in our meta-analysis used plasma or serum to measure ApoE levels, however, no studies used exactly the same assay method, further contributing to subject heterogeneity. Publication bias also seemed to influence our present results supporting the lower ApoE levels in AD. Asymmetrical missing data on the right side of the funnel plot suggested that studies failing to find the association between lower ApoE levels and AD may not have been reported or published, and we did not search for unpublished studies for original data. The large differences between published in 2001 or earlier and published after 2001 also implied publication bias.

To date, the underlying mechanisms involved in the association between ApoE protein and AD are uncertain. ApoE is an important modulator of plasma lipid metabolism and cholesterol homeostasis [10]. Previous studies showed that disturbances in cholesterol metabolism may influence synapse formation, function and stability. ApoE also serves as essential component in cholesterol delivery to neurons [37]. A lower level of plasma ApoE may impair the normal physiological functions of ApoE, contributing to cognitive decline and degeneration of CNS. Besides, ApoE is suggested to bind Aβ and promote its clearance and degradation, which is a decisive event in the pathogenesis of AD. A lower ApoE level may reduce the efficiency of Aβ clearance, and lead to the pathogenesis of AD [38]. Cramer et al. demonstrated that Aβ plaque and cognitive function were rapidly ameliorated in AD mouse models after treatment by an agonist that regulates ApoE expression [39]. In line with our results, many of those studies that did not meet the strict inclusion criteria for the meta-analysis also found a positive association between lower ApoE levels and AD. Van Vliet P et al. reported that offspring with a parental history of AD had lower plasma ApoE levels than subjects without a history [40]. By contrast, Kevin Taddei and his colleagues found increased plasma ApoE levels in AD groups compared to the controls [16]. Such inconsistent results might be related to confounding factors interfering with different assay protocols, sample size and demographic characteristics, which have not been fully elucidated. These studies were not directly comparable with our meta-analysis data because of lacking controls group, original data or diagnostic criteria for AD.

Our meta-analysis still had a lot of potential limitations. Firstly, significant heterogeneity existed overall. To clarify the sources of heterogeneity, we conducted the subgroup and sensitivity analyses. However, residual confounding factors across studies remain as a cause for concern in this meta-analysis. Therefore, heterogeneity was still an inevitable problem that may affect the precision of overall results. Secondly, few studies had reported gender specific ApoE levels, and we cannot identify whether ApoE level was significantly decreased in AD cases in both men and women. Thirdly, only 2 studies involving North American and one study involving Asia were included in this meta-analysis. More studies are needed from other countries to evaluate the association between ApoE levels and AD.

Nonetheless, results of our meta-analysis confirm that AD is associated with decreased levels of ApoE in peripheral blood, with the present of eight observational studies. While quantifying plasma ApoE does not seem particularly useful in AD diagnosis because of the various ApoE levels among the included studies. The preliminary and cross-sectional nature of the findings, the absence of assay standardization, must be addressed in future studies testing the usefulness of ApoE levels for clinical purposes. Herein, further-designed prospective studies with standardized assay method are also warranted to investigate potential roles of ApoE in the pathophysiology of AD, and potential benefits of ApoE supplementation in AD patients.

## Supporting Information

Checklist S1 **PRISMA 2009 checklist in this meta-analysis.**
(DOC)
